# Perioperative Adjunctive Esketamine for Postpartum Depression Among Women Undergoing Elective Cesarean Delivery: A Randomized Clinical Trial

**DOI:** 10.1001/jamanetworkopen.2024.0953

**Published:** 2024-03-06

**Authors:** Yu Chen, Yu Guo, Han Wu, Yi-Jie Tang, Suren Rao Sooranna, Li Zhang, Ting Chen, Xi-Yuan Xie, Liang-Cheng Qiu, Xiao-Dan Wu

**Affiliations:** 1 Department of Anesthesiology, Shengli Clinical Medical College of Fujian Medical University, Fuzhou, Fujian, China; 2 Department of Anesthesiology, Fujian Provincial Hospital, Fuzhou, Fujian, China; 3 Department of Metabolism, Digestion and Reproduction, Imperial College London, Chelsea and Westminster Hospital, London, United Kingdom; 4 Life Science and Clinical Research Center, Youjiang Medical University for Nationalities, Baise, China; 5 Department of Anesthesiology, Fujian Maternity and Child Health Hospital, Fuzhou, Fujian, China

## Abstract

**Importance:**

Postpartum depression (PPD) is one of the most common mental health conditions during the perinatal and postpartum periods, which can have adverse effects on both mother and infant.

**Objective:**

To investigate the efficacy of perioperative adjunctive esketamine administration after cesarean deliveries in the prevention of PPD.

**Design, Setting, and Participants:**

A single-center, double-blind, placebo-controlled, randomized clinical trial was conducted from January 1, 2022, to January 1, 2023, at Fujian Provincial Hospital among 298 women aged 18 to 40 years, with an American Society of Anesthesiologists grade I to III classification and singleton full-term pregnancies who were scheduled for elective cesarean deliveries. Primary analyses were performed on a modified intention-to-treat basis.

**Interventions:**

Patients were randomly assigned to the esketamine (n = 148) and control (n = 150) groups. Those in the esketamine group received a single intravenous injection of 0.25 mg/kg of esketamine immediately after fetal delivery, followed by 50 mg of esketamine as an adjuvant in patient-controlled intravenous analgesia for 48 hours after surgery. Saline was given to the control group of patients.

**Main Outcomes and Measures:**

The primary outcome was assessments of PPD symptoms by using the Edinburgh Postnatal Depression Scale (EPDS) at postpartum day 7. Positive screening for PPD was defined as a score of 10 or more points on the EPDS. In addition, the EPDS was analyzed as a continuous variable to evaluate depressive symptoms. Secondary outcomes included the Numeric Rating Scale (NRS) of postoperative pain, along with safety evaluations including adverse events and clinical assessments at postpartum days 14, 28, and 42.

**Results:**

A total of 298 pregnant women were included, with 150 in the control group (median age, 31.0 years [IQR, 29.0-34.0 years]) and 148 in the esketamine group (median age, 31.0 years [IQR, 28.0-34.0 years]). The prevalence of depression symptoms was significantly lower among patients given esketamine compared with controls (23.0% [34 of 148] vs 35.3% [53 of 150]; odds ratio, 0.55; 95% CI, 0.33-0.91; *P* = .02) on postpartum day 7. In addition, the esketamine group also showed a significantly lower change in EPDS scores (difference of least-squares means [SE], −1.17 [0.44]; 95% CI, −2.04 to −0.31; effect size, 0.74; *P* = .008). However, there were no differences between the groups in the incidence of positive screening results for PPD or in changes from the baseline EPDS scores at postpartum days 14, 28, and 42. There were no differences in NRS scores at rest and on movement except on movement at 72 hours postoperatively, when scores were significantly lower in the esketamine group (median, 3.0 [IQR, 2.0-3.0] vs 3.0 [IQR, 3.0-3.5]; median difference, 0 [95% CI, 0-0]; *P* = .03).

**Conclusions and Relevance:**

These results suggest that intravenous administration of esketamine during the perioperative period of elective cesarean delivery can improve depression symptoms during the early postpartum period. However, this antidepression effect may not be universally applicable to patients with low EPDS scores.

**Trial Registration:**

Chinese Clinical Trial Registry Identifier: ChiCTR2100054199

## Key Points

**Question:**

Does perioperative administration of adjunctive esketamine during cesarean delivery prevent postpartum depression?

**Findings:**

In this randomized clinical trial of 298 pregnant women who underwent elective cesarean delivery, intravenous administration of esketamine significantly decreased the incidence of screened positivity for postpartum depression and improved depressive symptoms during the early postpartum period.

**Meaning:**

This study suggests that perioperative adjunctive esketamine during cesarean delivery mitigates depressive symptoms, although the effect is transient and preoperative assessment of the mental status of patients should be a concern.

## Teaser

This randomized clinical trial investigates the efficacy of perioperative adjunctive esketamine administration after cesarean deliveries in the prevention of postpartum depression.

## Introduction

Postpartum depression (PPD) is a prevalent major depressive disorder that occurs in women during the puerperal period; it represents the most common mental health condition during both the perinatal and postpartum periods. The global incidence of PPD ranges from 15% to 30%,^1,2^ and in China approximately 25% of new mothers may be prone to PPD.^3,4^ Postpartum depression is characterized by a range of symptoms, including low mood, reduced interest in activities, sadness, irritability, insomnia, burnout, decreased attention, and even recurrent suicidal tendencies.^5^ These manifestations can influence the physical and psychological well-being of new mothers. Fourteen US Maternal Mortality Review Committees recently identified maternal mental health conditions as being responsible for 68% of pregnancy-related deaths, with PPD being the primary contributing factor.^6^ Furthermore, PPD may also adversely affect infants’ behavior, emotions, and cognition development.^7,8,9,10^ Such disorders can impose an additional burden to the mother and newborn as well as the entire family unit. These findings have encouraged researchers to search for possible remedies for PPD.

There is compelling evidence to link the glutamatergic system to depressive disorders, and excessive glutamate release can aggravate neural circuit injuries and functional impairment in mood regulation. There is a positive correlation between glutamate concentrations in crucial brain regions and PPD in new mothers when compared with controls.^11,12^ *N*-methyl-d-aspartate (NMDA) receptors have been implicated in glutamatergic neurotransmitter dysregulation^13,14^; these receptors may be a critical causative feature of mood disorders.^15,16^ Not only can ketamine, a classic uncompetitive glutamatergic NMDA receptor antagonist, block NMDA receptors directly, but it also inhibits the γ-aminobutyric acid–ergic interneuron activity and stimulates synaptogenesis,^17,18^ enabling it to provide antidepressant effects as well as acting as an anesthetic and analgesic.^19^ Several clinical trials have demonstrated that ketamine delivers rapid and effective antidepressant actions in patients with depression.^20,21,22^ It has also been shown to have a potential role in the treatment of PPD.^23^

Recently, esketamine, a novel antagonist of the NMDA receptor with a higher affinity than ketamine, has been used in several countries, including China. As a dextral resolution of ketamine, esketamine has been shown to improve the effectiveness of anesthetics and analgesics, with a similar antidepressant effect to its predecessor.^24,25^ Numerous studies have found that an esketamine nasal spray could rapidly induce and sustain beneficial effects for patients with a diagnosis of treatment-resistant depression and risk of suicides.^26,27,28,29^ Previous studies have also found that, for women who have undergone cesarean deliveries, a preventive administration of esketamine, either by the intrathecal or intravenous route, could yield both safe and efficacious analgesic effects.^30,31^ Given its bidirectional efficacy as an analgesic and antidepressant, we performed a double-blind, randomized clinical trial to investigate the effects of perioperative adjunctive esketamine administration during cesarean delivery on patients with PPD.

## Methods

### Study Design

This prospective, double-blind, placebo-controlled randomized clinical trial was approved by the Institutional Ethics Committee of Fujian Provincial Hospital and registered in the Chinese Clinical Trials Registry (ChiCTR2100054199). The trial protocol and statistical analysis plan are provided in Supplement 1. Patients were enrolled between January 1, 2022, and January 1, 2023. All participants provided written informed consent to participate in this study, which followed the Consolidated Standards of Reporting Trials (CONSORT) reporting guideline.

We enrolled 18- to 40-year-old pregnant women with an American Society of Anesthesiologists (ASA) grade I to III classification and a singleton pregnancy at full term (>37 weeks). They were scheduled for elective cesarean deliveries in either the Fujian Provincial Hospital or its southern branch. Patients excluded were those with prenatal mental disorders, either hypertension or risk of intracranial hypertension, preeclampsia, eclampsia, hyperthyroidism, placenta previa, placental abruption, and placenta accreta. Others with an allergy to NMDA receptor antagonists and an inability or unwillingness to cooperate with questionnaires and clinical examinations were also excluded.

### Randomization and Blinding

Patients were randomly assigned in a 1:1 ratio into 2 groups, the esketamine and control groups, using a computerized randomization table. Before anesthesia, the study coordinator opened the envelopes consecutively according to the recruitment sequence and the drugs to be used for each patient were prepared. All patients, anesthesiologists, surgeons, follow-up interviewers, and investigators who were involved in data collection and analysis were blinded to the allocation quota of each group throughout the study period (eFigure in Supplement 2).

### Perioperative Anesthesia and Management of Analgesia

After the patient entered the operating room, an intravenous channel was established, and the patient’s pulse oximetry, electrocardiograph, and noninvasive blood pressure were monitored. Oxygen was provided at 2 L/min through a nasal catheter. The patient was laid in a left lateral position and a spinal needle was punctured into the subarachnoid space through the L2-L3 or L3-L4 interspace. Then 12 mg of 0.6% ropivacaine was administered. After withdrawing the needle, a reinforced epidural catheter was placed 3 to 4 cm into the epidural space; 5 mL of 2% lidocaine was then administered through the catheter, which was used to assess the effect of the anesthetic. The upper sensory block level was adjusted to between the T6 and T7 levels. While monitoring any changes in blood pressure, if necessary, the patient was repositioned onto a left lateral tilt of 30° and 6 to 12 mg of ephedrine and colloidal solutions for hemodynamic stabilization were intravenously administered. Immediately after delivery of the fetus, the patients in the esketamine group received 0.25 mg/kg of esketamine and 2 mg of midazolam intravenously, which were diluted to a volume of 10 mL with 0.9% saline. An equal volume of normal saline and 2 mg of midazolam were injected for patients in the control group. Then, a patient-controlled intravenous analgesia device was used to administer a mixture of 100 μg of sufentanil, 50 mg of esketamine, and 0.25 mg of palonosetron hydrochloride in 100 mL of saline. For the patients in the control group, only 100 μg of sufentanil and 0.25 mg of palonosetron hydrochloride in 100 mL of saline were given via a patient-controlled intravenous analgesia pump. The pumps were set to a background infusion rate of 2 mL/h for 48 hours. A bolus dose of 2 mL was given and a lockout time of 10 minutes was used in both groups.

### Outcome Measurements

The baseline data encompassed demographic characteristics, ASA classification, number of deliveries, gestational duration, and pregestational comorbidities. Intraoperative data consisted of duration of surgery, intraoperative bleeding, and vasopressor use. Neonatal data included newborn weight, Apgar scores assessed at 1 and 5 minutes after birth, umbilical arterial blood gas values, and the neonatal intensive care unit admission rate.

Our primary outcome was the self-report of postpartum depressive symptoms, which were assessed using the Edinburgh Postnatal Depression Scale (EPDS).^32^ This scale is a widely used postpartum depression screening test.^33^ It consists of 10 items, each of which is scored on a 4-point scale (range, 0-3). Total EPDS scores, which ranged from 0 to 30 points, were the sum of the scores from each item. To determine the incidence of PPD, a cutoff of 10 or more points was defined as positive for PPD according to the published recommendations.^34,35,36^ Furthermore, to evaluate depressive symptoms among the 2 cohorts, we also analyzed the EPDS score as a continuous variable. This involved comparing the change in this parameter between the 2 groups from baseline (the day before surgery) to the end points. Our primary efficacy end point was set at postpartum day 7, and secondary efficacy end points were assessed at days 14, 28, and 42 after delivery. The scale evaluation was performed by a trained interviewer (T.C.) who was blinded to the protocol details and group allocation.

Our secondary outcomes included maternal pain intensity at rest as well as during movements and were evaluated at 12, 24, 48, and 72 hours after surgery, in addition to postpartum pain sensitivity on postpartum day 7. The assessment of pain intensity was conducted using the Numeric Rating Scale (NRS), an 11-point scale ranging from 0 to 10, where 0 represents no pain and 10 represents the most severe pain. Furthermore, the recovery of postoperative gastrointestinal function was estimated by recording the times to first flatus and defecation. We also monitored all postoperative complications within 3 days of surgery, including nausea and vomiting, as well as neuropsychiatric symptoms (such as nystagmus, dizziness, headaches, nightmares, and hallucinations).

### Statistical Analysis

The estimated sample size was calculated using PASS software, version 15.0 (NCSS), and this was based on our preliminary study, in which the incidence of screened positivity for PPD on postpartum day 7 was 35% for patients who underwent cesarean delivery. The expected effect size was subsequently calculated to detect a 50% prevalence reduction in PPD after surgery, with a 2-sided α = .05 and 90% power. The sample size was determined to be 128 patients. After loss to follow-up and consent withdrawals, 150 patients were selected for inclusion in each arm of this trial.

Statistical analysis was performed using SPSS, version 26.0 (IBM SPSS) and R statistical software, version 4.2.0 (R Project for Statistical Computing). The normality of variables was determined using the Kolmogorov-Smirnov test. Normally distributed continuous data were presented as mean (SD) values, and intergroup comparisons used either the unpaired 2-tailed *t* test or 1-way analysis of variance. Nonnormally distributed variables were presented as median (IQR) values, and intergroup comparisons were determined using the Mann-Whitney test. Percentages were used to present the categorical variables; these were compared using either the χ^2^ test or the Fisher exact test. The changes in EPDS scores at different time points between the groups were analyzed with a mixed-effect model using repeated measures. This model included the baseline EPDS score as the covariate, and treatment day, as well as day-by-treatment interactions, as fixed effects. Random intercepts and unstructured covariance structures were used to model the within-patient errors. An exploratory analysis was conducted to evaluate the disparities in our primary outcomes within the predetermined subgroups. The stratification confounders were corrected by using the Cochran-Mantel-Haenszel test. Primary analyses were performed by a modified intention-to-treat analysis set, which included all randomly assigned participants who received treatment and had at least 1 PPD screening assessment.^37^ A *P* < .05 was considered to be statistically significant. The findings for secondary outcomes and subgroup analyses should be considered exploratory due to the potential for type I errors resulting from multiple comparisons.

## Results

**Figure 1.**
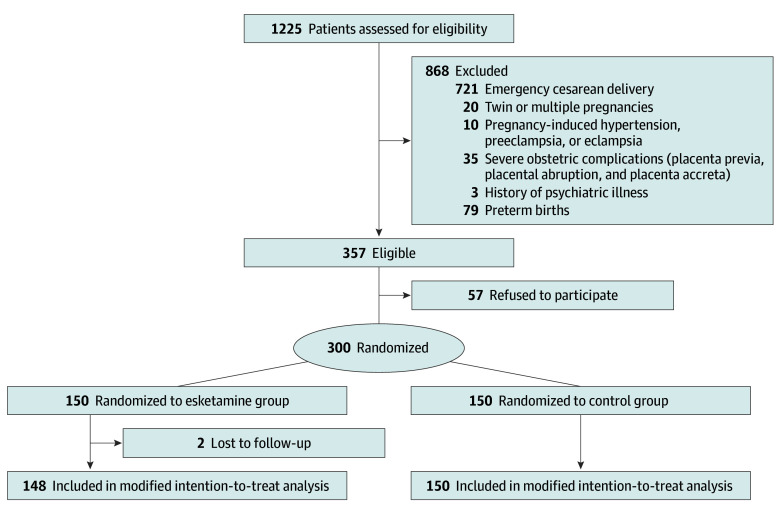
CONSORT Diagram Representing the Protocol for Patients in This Study

A total of 1225 pregnant women were assessed for eligibility between January 1, 2022 and January 1, 2023. Of these, 357 were eligible, and 150 each were enrolled and randomly assigned to the esketamine and control groups. Among the enrolled patients, 2 women refused follow-up at postpartum day 5, and 298 women were included in the modified intention-to-treat analysis (148 in the esketamine group [median age, 31.0 years (IQR, 28.0-34.0 years)] and 150 in the control group [median age, 31.0 years (IQR, 29.0-34.0 years)]; Figure 1). Baseline characteristics, intraoperative data, and neonatal outcomes were well balanced between the 2 groups (Table). Most patients were classified as ASA class II (esketamine group, 97.3% [144 of 148]; control group, 96.0% [144 of 150]) and multipara (esketamine group, 66.9% [99 of 148]; control group, 63.3% [95 of 150]). The mean (SD) baseline EPDS scores were 4.2 (2.3) and 4.0 (2.2) in the esketamine and control groups, respectively.

**Table.** Baseline Characteristics and Intraoperative Variables of the Patients and Neonatal Outcomes^a^

Variable	Esketamine group (n = 148)	Control group (n = 150)
Baseline data		
Age, median (IQR), y	31.0 (28.0-34.0)	31.0 (29.0-34.0)
ASA classification, No. (%)		
I	4 (2.7)	6 (4.0)
II	144 (97.3)	144 (96.0)
III	0	0
Primipara, No. (%)	49 (33.1)	55 (36.7)
Cause of elective cesarean delivery, No. (%)		
Malpresentation	46 (31.1)	49 (32.7)
Scarred uterus	93 (62.8)	91 (60.7)
Suspected fetal macrosomia	5 (3.4)	5 (3.3)
CDMR	4 (2.7)	5 (3.3)
Weight, mean (SD), kg	70.1 (8.8)	68.6 (9.4)
Height, mean (SD), cm	159.5 (5.3)	159.5 (5.9)
Prenatal BMI, mean (SD)	27.5 (3.1)	26.9 (3.3)
Duration of gestation, median (IQR), d	275.0 (270.0-279.0)	275.5 (271.0-279.0)
Comorbidities, No. (%)		
GDM	28 (18.9)	28 (18.7)
Anemia	8 (5.4)	5 (3.3)
Hyperlipidemia	59 (39.9)	52 (34.7)
Hypothyroidism	3 (2.0)	0
Baseline EPDS score, mean (SD)	4.2 (2.3)	4.0 (2.2)
Intraoperative variables		
Duration of surgery, mean (SD), min	113.8 (9.2)	111.2 (8.7)
Estimated blood loss, mean (SD), mL	366.8 (137.1)	371.5 (137.6)
Dose of oxytocin, mean (SD), μg	95.0 (19.9)	96.0 (26.9)
Use of vasopressors, No. (%)	73 (49.3)	68 (45.3)
Neonatal outcomes		
Birth weight, mean (SD), g	3359.5 (440.5)	3364.5 (416.3)
Apgar scores, median (IQR)		
1 min	10.0 (10.0-10.0)	10.0 (10.0-10.0)
5 min	10.0 (10.0-10.0)	10.0 (10.0-10.0)
10 min	10.0 (10.0-10.0)	10.0 (10.0-10.0)
Umbilical arterial blood gas values		
pH, mean (SD)	7.1 (0.2)	7.1 (0.3)
Glucose, mean (SD), mg/dL	95.4 (25.2)	97.2 (30.6)
Lactate, mean (SD), mmol/L	6.2 (2.2)	6.5 (2.3)
Transferred to NICU, No. (%)^b^	13 (8.8)	15 (10.0)

Abbreviations: ASA, American Society of Anesthesiologists; BMI, body mass index (calculated as weight in kilograms divided by height in meters squared); CDMR, cesarean delivery on maternal request; EPDS, Edinburgh Postnatal Depression Scale (range, 0-30; a cutoff of ≥10 points was defined as a positive screening result for postpartum depression); GDM, gestational diabetes mellitus; NICU, neonatal intensive care unit.

SI conversion factors: To convert glucose to millimoles per liter, multiply by 0.0555.

^a^ Data presented as mean (SD) values were compared using the unpaired, 2-tailed *t* test. Data presented as median (IQR) values were compared using the Mann-Whitney test. Data reported as the number (percentage) of patients were compared using either the χ^2^ test or the Fisher exact test.

^b^ Needed by the pediatricians for further monitoring and/or treatment of patients. Indications included low Apgar scores (<7), neonatal acidosis, neonatal hypoglycemia, neonatal malformation, and premature delivery.

### Efficacy Outcomes

**Figure 2.**
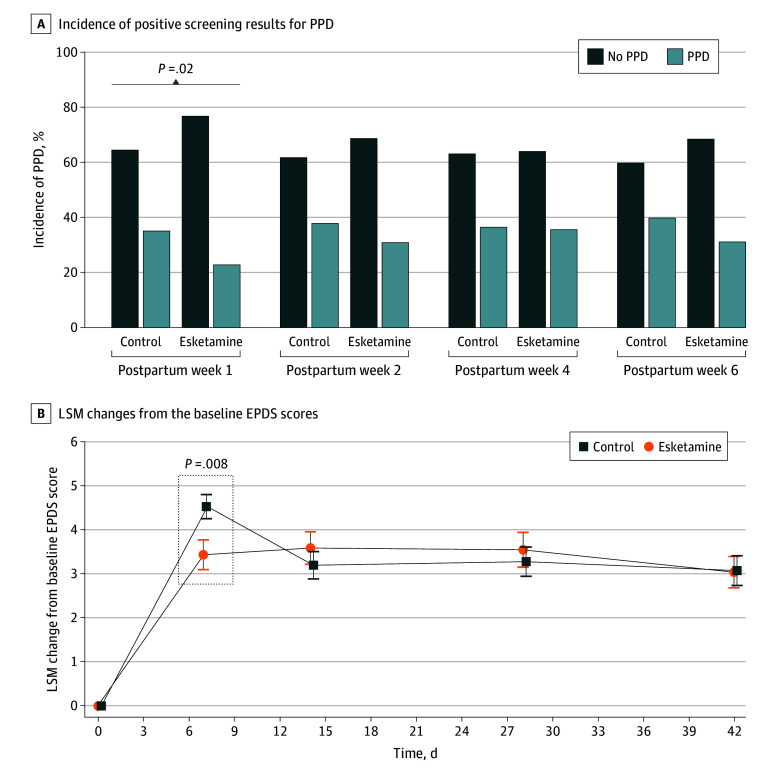
Incidence of Positive Screening Results for Postpartum Depression (PPD) and Least-Square Mean (LSM) Changes From the Baseline Edinburgh Postnatal Depression Scale (EPDS) Scores A, Compared with the control group, the esketamine group showed a significant decrease in the incidence of positivity screening results for PPD at 7 days post partum, with no significant differences observed at the rest of the time points. B, Significant enhancements in depressive symptoms, as evaluated by the EPDS scores, were observed at 7 days post partum in the esketamine group compared with the control group. However, no statistically significant differences were found between the 2 groups at any subsequent time points.

At the primary end point, the prevalence of PPD was significantly lower for patients assigned to the esketamine group (34 of 148 [23.0%]) compared with the control group (53 of 150 [35.3%]; odds ratio, 0.55; 95% CI, 0.33-0.91; *P* = .02) (eTable 1 in Supplement 2). In addition, a significant decrease was observed in the extent of EPDS score elevation from baseline at postpartum day 7 in the esketamine group compared with the control group (least-squares means difference [LSMD] [SE], −1.17 [0.44]; 95% CI, −2.04 to −0.31; effect size, 0.74; *P* = .008). However, there were no meaningful differences between the groups in PPD incidence in the other efficacy end points (day 14: 46 of 148 [31.1%] vs 57 of 150 [38.0%]; *P* = .23; day 28: 53 of 148 [35.8%] vs 55 of 150 [36.7%]; *P* = .90; day 42: 50 of 148 [33.8%] vs 56 of 150 [37.3%]; *P* = .55) (eTable 1 in Supplement 2; Figure 2A). With respect to the antidepression effects of esketamine subsiding over time, no significant differences were noted in least-squares mean between-group differences at day 14 (LSMD [SE], 0.32 [0.49]; 95% CI, −0.63 to 1.28; *P* = .51), day 28 (LSMD [SE], 0.24 [0.52]; 95% CI, −0.78 to 1.26; *P* = .64), and day 42 (LSMD [SE], –0.06 [0.49]; 95% CI, −1.03 to 0.91; *P* = .90) (eTable 2 in Supplement 2; Figure 2B).

**Figure 3.**
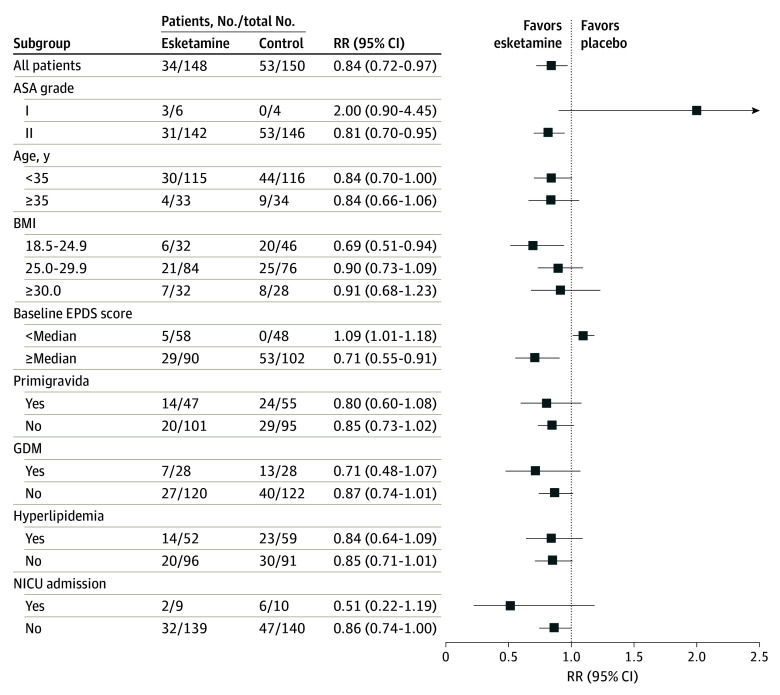
Forest Plot Assessing the Effects of Esketamine vs Control in the Predefined Subgroups ASA indicates American Society of Anesthesiologists; BMI, body mass index (calculated as weight in kilograms divided by height in meters squared); EPDS, Edinburgh Postnatal Depression Scale; GDM, gestational diabetes mellitus; NICU, neonatal intensive care unit; and RR, relative risk.

**Figure 4.**
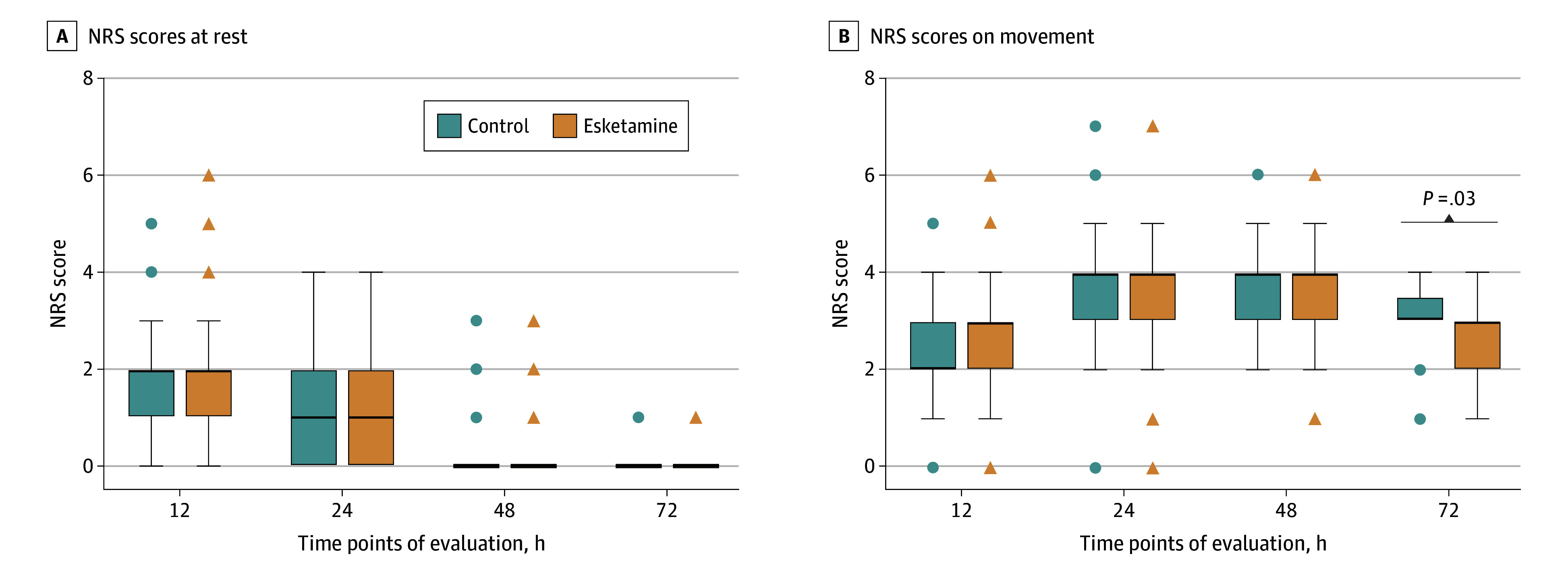
Numeric Rating Scale (NRS) Pain Scores Between the 2 Study Groups The NRS is an 11-point scale, with 0 and 10 indicating no pain and the worst pain, respectively. A, Comparison of the NRS value at rest between the 2 groups. B, Comparison of the NRS value on movement between the 2 groups. The box and whisker plots show the median values, IQRs, and outlier values, with the circles and triangles indicating outliers for the control and esketamine groups, respectively.

For subgroups of more than 15 patients, all the primary end points analyzed demonstrated a favorable advantage toward esketamine, except for patients with baseline EPDS scores lower than the median (relative risk, 1.09; 95% CI, 1.01-1.18; *P* = .05; Figure 3). However, the NRS pain scores for movements at 72 hours postoperatively were significantly lower in the esketamine group compared with the control group (median, 3.0 [IQR, 2.0-3.0] vs 3.0 [IQR, 3.0-3.5]; median difference, 0 [95% CI, 0-0]; *P* = .03), although there were no differences on movements observed at the other time points (eTable 3 in Supplement 2). In addition, the NRS scores at rest were similar at all measured time points (eTable 3 in Supplement 2; Figure 4). The mean times to first flatus and first defecation were 1 day and 2 days, respectively, for both groups (eTable 4 in Supplement 2). The mean length of hospital stay, which was 4 days, was also not different between the groups.

### Safety Outcomes

During the 3 days after surgery, esketamine was generally well tolerated. There was no difference in the incidence of nausea and vomiting between the 2 groups. In addition, mothers in the esketamine group did not demonstrate any significant differences in mental symptoms, including dizziness, headaches, somnolence, hallucinations, nightmares, and nystagmus, compared with the control group (eTable 5 in Supplement 2).

## Discussion

In this randomized clinical trial, we observed that intravenous administration of esketamine during the perioperative period of elective cesarean delivery can provide significant and short-term reduction in depressive symptoms during the initial postpartum period. However, the aforementioned effects tended to diminish quickly with time.

Both preclinical and clinical studies have found that NMDA receptor abnormalities play a role in several psychiatric diseases, especially during depression disorders, by altering glutamatergic neurotransmission.^16,38,39^ Therefore, the roles of NMDA receptor antagonists in the prevention of PPD have attracted increasing attention. A recent meta-analysis of clinically controlled trials of intravenous administration of a subanesthetic dose of ketamine during the cesarean delivery period in PPD reported that a dose of 0.5 mg/kg or more might help in reducing the prevalence of PPD within 1 week after delivery, but had no beneficial effect after 4 weeks post partum.^40^ Esketamine, a novel NMDA receptor antagonist, is generally considered to have more potent efficacy among antidepressants.^41^ In the present study, we found that a single dose of 0.5 mg/kg of esketamine during cesarean delivery, followed by continuous intravenous administration of low-dose esketamine, 1.0 mg/h, for 48 hours can significantly reduce depressive symptoms associated with PPD within 7 days after delivery. However, this effect rapidly disappeared after withdrawal of the esketamine treatment.

Our findings were similar to previous studies that reported that combined intravenous administration of esketamine during the perioperative period could achieve a rapid and short-term antidepressive effect in women undergoing cesarean delivery.^42,43^ However, some studies have shown inconsistent results of the antidepressant effect of esketamine on patients undergoing cesarean delivery. A recent randomized clinical trial involving 150 patients undergoing elective cesarean delivery showed that perioperative use of esketamine might not help in reducing the incidence of PPD among new mothers.^44^ Moreover, another study also found that a single intravenous injection of low-dose esketamine was unable to reduce the prevalence of depression after birth.^45^ These discrepancies may be due to differences in the dose, mode, and timing of therapeutic administration.

Unexpectedly, our subgroup analysis discovered that patients with low baseline EPDS scores in the control group had greater reduction in depressive symptoms than those in the esketamine group. These results emphasize that esketamine may not be administered to all women in childbirth. A preclinical study performed on male rhesus monkeys showed that a single administration of intravenous esketamine could induce the release of dopamine in the striatum.^46^ In addition, esketamine-mediated presynaptic dopamine release can induce some psychotomimetic and dissociative effects in healthy individuals.^47^ A randomized crossover trial in healthy control individuals also showed that ketamine could elicit depressive symptoms, specifically anhedonia.^48^ Collectively, these data suggest that caution is warranted with using esketamine in women during cesarean delivery without prior evidence of prenatal depression, and the importance of a prenatal mental status assessment for patients undergoing elective cesarean delivery is emphasized.

The pathophysiology of PPD is still unclear,^49^ and its onset and progression may be associated with perinatal pain.^50^ Lim et al^51^ linked labor analgesia to PPD, and found that adequate perinatal analgesia was an effective strategy for reducing its incidence. Moreover, a previous prospective cohort study stratified parturient women who received labor analgesia based on their individual preferences and found that the incidence of PPD increased significantly among women who received unplanned emergency labor analgesia due to their inability to tolerate pain.^52^ This finding indicated that perinatal pain may affect the postpartum mental state, thereby contributing to the development of PPD. Whereas, we found, as in previous studies, that adjunctive esketamine administration during the perioperative period could not effectively improve pain symptoms for up to 72 hours postoperatively.^23,44,53^ Accordingly, it was presumed that the antidepressant effect of esketamine on postpartum women might not be related to its analgesic property. However, further studies are required to confirm the role of antihyperalgesics in the preventive effect of esketamine on PPD. In addition, there were no notable neurologic symptoms observed during the postoperative period in the esketamine group, in line with previous reports.^31,44^

### Limitations

This study has some limitations. We evaluated the influence of esketamine only on PPD, and some other potential confounding factors of PPD were not collected, including socioeconomic status, emotional support from the spouse and extended family, stressful life events, and other negative stressful events. These factors may have affected our results. Second, considering that the peak time for PPD to manifest is approximately 42 days post partum,^54^ we set our follow-up end at this time point. However, the long-term sustainability of any treatment response beyond this specified period remains uncertain, and needs to be clarified. Third, this trial was conducted at 2 sites within a single center, and our participants were limited to Chinese new mothers. Therefore, the findings may lack generalizability to wider global populations and multicenter studies, involving different regions, countries, and races and ethnicities, are needed.

## Conclusions

This randomized clinical trial showed that an intravenous injection of esketamine during the perioperative period of elective cesarean delivery was effective in improving depressive symptoms in the early postpartum period, although the effect faded rapidly over time. We also found that this antidepression effect may not be generalized to all patients because those with low baseline EPDS scores performed better in the absence of esketamine. Moreover, the administration of esketamine to the patients demonstrated a satisfactory level of safety and tolerability. Overall, our study demonstrated a potential beneficial effect of perioperative adjunctive esketamine administration on the treatment of PPD in women undergoing elective cesarean deliveries.

## Notes

Supplement 1.
Trial Protocol and Statistical Analysis Plan

Supplement 2. **eTable 1.** The Prevalence of Women With a Positive PPD Screening (EPDS Score ≥10) Between the Two Groups **eTable 2.** The Changes in EPDS Scores From Baseline Values to the End-Points of the Trial Period Between the Two Groups **eTable 3.** The Numeric Rating Scale Pain Scores Between the Two Groups at Different Time Points **eTable 4.** Postoperative Recovery Outcomes Between the Two Groups **eTable 5.** Adverse Events in the Study Participants **eFigure.** Trial Intervention Diagram

Supplement 3.
Data Sharing Statement

## References

[1] Liu Y, Zhang L, Guo N, Jiang H. Postpartum depression and postpartum post-traumatic stress disorder: prevalence and associated factors. BMC Psychiatry. 2021;21(1):487. doi:10.1186/s12888-021-03432-7 34610797 PMC8491367

[2] Deligiannidis KM, Meltzer-Brody S, Gunduz-Bruce H, . Effect of zuranolone vs placebo in postpartum depression: a randomized clinical Trial. JAMA Psychiatry. 2021;78(9):951-959. doi:10.1001/jamapsychiatry.2021.1559 34190962 PMC8246337

[3] Liu X, Wang S, Wang G. Prevalence and risk factors of postpartum depression in women: a systematic review and meta-analysis. J Clin Nurs. 2022;31(19-20):2665-2677. doi:10.1111/jocn.16121 34750904

[4] Wang Z, Liu J, Shuai H, . Mapping global prevalence of depression among postpartum women. Transl Psychiatry. 2021;11(1):543. doi:10.1038/s41398-021-01663-6 34671011 PMC8528847

[5] Stewart DE, Vigod SN. Postpartum depression: pathophysiology, treatment, and emerging therapeutics. Annu Rev Med. 2019;70:183-196. doi:10.1146/annurev-med-041217-011106 30691372

[6] Trost SL, Beauregard JL, Smoots AN, . Preventing pregnancy-related mental health deaths: insights from 14 US Maternal Mortality Review Committees, 2008-17. Health Aff (Millwood). 2021;40(10):1551-1559. doi:10.1377/hlthaff.2021.00615 34606354 PMC11135281

[7] Feldman R, Granat A, Pariente C, Kanety H, Kuint J, Gilboa-Schechtman E. Maternal depression and anxiety across the postpartum year and infant social engagement, fear regulation, and stress reactivity. J Am Acad Child Adolesc Psychiatry. 2009;48(9):919-927. doi:10.1097/CHI.0b013e3181b21651 19625979

[8] Becker M, Weinberger T, Chandy A, Schmukler S. Depression during pregnancy and postpartum. Curr Psychiatry Rep. 2016;18(3):32. doi:10.1007/s11920-016-0664-7 26879925

[9] Al Nasr RS, Altharwi K, Derbah MS, . Prevalence and predictors of postpartum depression in Riyadh, Saudi Arabia: a cross sectional study. PLoS One. 2020;15(2):e0228666. doi:10.1371/journal.pone.0228666 32040495 PMC7010279

[10] Weissman MM. Postpartum depression and its long-term impact on children: many new questions. JAMA Psychiatry. 2018;75(3):227-228. doi:10.1001/jamapsychiatry.2017.4265 29387871

[11] Moriguchi S, Takamiya A, Noda Y, . Glutamatergic neurometabolite levels in major depressive disorder: a systematic review and meta-analysis of proton magnetic resonance spectroscopy studies. Mol Psychiatry. 2019;24(7):952-964. doi:10.1038/s41380-018-0252-9 30315224 PMC6755980

[12] Rosa CE, Soares JC, Figueiredo FP, . Glutamatergic and neural dysfunction in postpartum depression using magnetic resonance spectroscopy. Psychiatry Res Neuroimaging. 2017;265:18-25. doi:10.1016/j.pscychresns.2017.04.008 28494346

[13] Uno Y, Coyle JT. Glutamate hypothesis in schizophrenia. Psychiatry Clin Neurosci. 2019;73(5):204-215. doi:10.1111/pcn.12823 30666759

[14] Jorratt P, Hoschl C, Ovsepian SV. Endogenous antagonists of *N*-methyl-d-aspartate receptor in schizophrenia. Alzheimers Dement. 2021;17(5):888-905. doi:10.1002/alz.12244 33336545

[15] Bhatia NY, Ved HS, Kale PP, Doshi GM. Importance of exploring *N*-methyl-d-aspartate (NMDA) as a future perspective target in depression. CNS Neurol Disord Drug Targets. 2022;21(10):1004-1016. doi:10.2174/1871527321666220329141639 35352638

[16] Wei Y, Chang L, Hashimoto K. Molecular mechanisms underlying the antidepressant actions of arketamine: beyond the NMDA receptor. Mol Psychiatry. 2022;27(1):559-573. doi:10.1038/s41380-021-01121-1 33963284 PMC8960399

[17] Li X, Saiyin H, Chen X, Yu Q, Ma L, Liang W. Ketamine impairs growth cone and synaptogenesis in human GABAergic projection neurons via GSK-3β and HDAC6 signaling. Mol Psychiatry. Published online November 21, 2022. doi:10.1038/s41380-022-01864-5 36414713 PMC11371642

[18] Zanos P, Gould TD. Mechanisms of ketamine action as an antidepressant. Mol Psychiatry. 2018;23(4):801-811. doi:10.1038/mp.2017.255 29532791 PMC5999402

[19] Subramanian S, Haroutounian S, Palanca BJA, Lenze EJ. Ketamine as a therapeutic agent for depression and pain: mechanisms and evidence. J Neurol Sci. 2022;434:120152. doi:10.1016/j.jns.2022.120152 35092901

[20] Opler LA, Opler MG, Arnsten AF. Ameliorating treatment-refractory depression with intranasal ketamine: potential NMDA receptor actions in the pain circuitry representing mental anguish. CNS Spectr. 2016;21(1):12-22. doi:10.1017/S1092852914000686 25619798 PMC4515405

[21] Phillips JL, Norris S, Talbot J, . Single, repeated, and maintenance ketamine infusions for treatment-resistant depression: a randomized controlled trial. Am J Psychiatry. 2019;176(5):401-409. doi:10.1176/appi.ajp.2018.18070834 30922101

[22] Smith-Apeldoorn SY, Veraart JK, Spijker J, Kamphuis J, Schoevers RA. Maintenance ketamine treatment for depression: a systematic review of efficacy, safety, and tolerability. Lancet Psychiatry. 2022;9(11):907-921. doi:10.1016/S2215-0366(22)00317-0 36244360

[23] Ma JH, Wang SY, Yu HY, . Prophylactic use of ketamine reduces postpartum depression in Chinese women undergoing cesarean section. Psychiatry Res. 2019;279:252-258. doi:10.1016/j.psychres.2019.03.026 31147085

[24] Bozymski KM, Crouse EL, Titus-Lay EN, Ott CA, Nofziger JL, Kirkwood CK. Esketamine: a novel option for treatment-resistant depression. Ann Pharmacother. 2020;54(6):567-576. doi:10.1177/1060028019892644 31795735

[25] Molero P, Ramos-Quiroga JA, Martin-Santos R, Calvo-Sánchez E, Gutiérrez-Rojas L, Meana JJ. Antidepressant efficacy and tolerability of ketamine and esketamine: a critical review. CNS Drugs. 2018;32(5):411-420. doi:10.1007/s40263-018-0519-3 29736744

[26] Singh JB, Fedgchin M, Daly E, . Intravenous esketamine in adult treatment-resistant depression: a double-blind, double-randomization, placebo-controlled study. Biol Psychiatry. 2016;80(6):424-431. doi:10.1016/j.biopsych.2015.10.018 26707087

[27] Daly EJ, Singh JB, Fedgchin M, . Efficacy and safety of intranasal esketamine adjunctive to oral antidepressant therapy in treatment-resistant depression: a randomized clinical trial. JAMA Psychiatry. 2018;75(2):139-148. doi:10.1001/jamapsychiatry.2017.3739 29282469 PMC5838571

[28] Popova V, Daly EJ, Trivedi M, . Efficacy and safety of flexibly dosed esketamine nasal spray combined with a newly initiated oral antidepressant in treatment-resistant depression: a randomized double-blind active-controlled study. Am J Psychiatry. 2019;176(6):428-438. doi:10.1176/appi.ajp.2019.19020172 31109201

[29] Kasper S, Cubała WJ, Fagiolini A, Ramos-Quiroga JA, Souery D, Young AH. Practical recommendations for the management of treatment-resistant depression with esketamine nasal spray therapy: basic science, evidence-based knowledge and expert guidance. World J Biol Psychiatry. 2021;22(6):468-482. doi:10.1080/15622975.2020.1836399 33138665

[30] Unlugenc H, Ozalevli M, Gunes Y, . A double-blind comparison of intrathecal S(+) ketamine and fentanyl combined with bupivacaine 0.5% for caesarean delivery. Eur J Anaesthesiol. 2006;23(12):1018-1024. doi:10.1017/S0265021506000950 16824240

[31] Xu LL, Wang C, Deng CM, . Efficacy and safety of esketamine for supplemental analgesia during elective cesarean delivery: a randomized clinical trial. JAMA Netw Open. 2023;6(4):e239321. doi:10.1001/jamanetworkopen.2023.9321 37083664 PMC10122167

[32] Cox JL, Holden JM, Sagovsky R. Detection of postnatal depression: development of the 10-item Edinburgh Postnatal Depression Scale. Br J Psychiatry. 1987;150:782-786. doi:10.1192/bjp.150.6.782 3651732

[33] Coll CVN, Domingues MR, Stein A, . Efficacy of regular exercise during pregnancy on the prevention of postpartum depression: the PAMELA randomized clinical trial. JAMA Netw Open. 2019;2(1):e186861. doi:10.1001/jamanetworkopen.2018.6861 30646198 PMC6324311

[34] Hinkle SN, Buck Louis GM, Rawal S, Zhu Y, Albert PS, Zhang C. A longitudinal study of depression and gestational diabetes in pregnancy and the postpartum period. Diabetologia. 2016;59(12):2594-2602. doi:10.1007/s00125-016-4086-1 27640810 PMC5101167

[35] Chen Y, Ye X, Wu H, . Association of postpartum pain sensitivity and postpartum depression: a prospective observational study. Pain Ther. 2021;10(2):1619-1633. doi:10.1007/s40122-021-00325-1 34580805 PMC8586323

[36] Luo SC, Duan KM, Fang C, . Correlations between *SIRT* genetic polymorphisms and postpartum depressive symptoms in Chinese parturients who had undergone cesarean section. Neuropsychiatr Dis Treat. 2020;16:3225-3238. doi:10.2147/NDT.S278248 33380799 PMC7769146

[37] Goodwin GM, Aaronson ST, Alvarez O, . Single-dose psilocybin for a treatment-resistant episode of major depression. N Engl J Med. 2022;387(18):1637-1648. doi:10.1056/NEJMoa2206443 36322843

[38] Murrough JW, Abdallah CG, Mathew SJ. Targeting glutamate signalling in depression: progress and prospects. Nat Rev Drug Discov. 2017;16(7):472-486. doi:10.1038/nrd.2017.16 28303025

[39] Hashimoto K, Bruno D, Nierenberg J, . Abnormality in glutamine-glutamate cycle in the cerebrospinal fluid of cognitively intact elderly individuals with major depressive disorder: a 3-year follow-up study. Transl Psychiatry. 2016;6(3):e744. doi:10.1038/tp.2016.8 26926880 PMC4872461

[40] Li Q, Wang S, Mei X. A single intravenous administration of a sub-anesthetic ketamine dose during the perioperative period of cesarean section for preventing postpartum depression: a meta-analysis. Psychiatry Res. 2022;310:114396. doi:10.1016/j.psychres.2022.114396 35278826

[41] Kaur U, Pathak BK, Singh A, Chakrabarti SS. Esketamine: a glimmer of hope in treatment-resistant depression. Eur Arch Psychiatry Clin Neurosci. 2021;271(3):417-429. doi:10.1007/s00406-019-01084-z 31745646

[42] Han Y, Li P, Miao M, Tao Y, Kang X, Zhang J. S-ketamine as an adjuvant in patient-controlled intravenous analgesia for preventing postpartum depression: a randomized controlled trial. BMC Anesthesiol. 2022;22(1):49. doi:10.1186/s12871-022-01588-7 35172727 PMC8848809

[43] Yang SQ, Zhou YY, Yang ST, . Effects of different doses of esketamine intervention on postpartum depressive symptoms in cesarean section women: a randomized, double-blind, controlled clinical study. J Affect Disord. 2023;339:333-341. doi:10.1016/j.jad.2023.07.007 37442447

[44] Liu QR, Zong QK, Ding LL, . Effects of perioperative use of esketamine on postpartum depression risk in patients undergoing cesarean section: a randomized controlled trial. J Affect Disord. 2023;339:815-822. doi:10.1016/j.jad.2023.07.103 37482224

[45] Shen J, Song C, Lu X, . The effect of low-dose esketamine on pain and post-partum depression after cesarean section: a prospective, randomized, double-blind clinical trial. Front Psychiatry. 2023;13:1038379. doi:10.3389/fpsyt.2022.1038379 36683972 PMC9845877

[46] Hashimoto K, Kakiuchi T, Ohba H, Nishiyama S, Tsukada H. Reduction of dopamine D_2/3_ receptor binding in the striatum after a single administration of esketamine, but not R-ketamine: a PET study in conscious monkeys. Eur Arch Psychiatry Clin Neurosci. 2017;267(2):173-176. doi:10.1007/s00406-016-0692-7 27091456 PMC5323469

[47] Bonaventura J, Lam S, Carlton M, . Pharmacological and behavioral divergence of ketamine enantiomers: implications for abuse liability. Mol Psychiatry. 2021;26(11):6704-6722. doi:10.1038/s41380-021-01093-2 33859356 PMC8517038

[48] Nugent AC, Ballard ED, Gould TD, . Ketamine has distinct electrophysiological and behavioral effects in depressed and healthy subjects. Mol Psychiatry. 2019;24(7):1040-1052. doi:10.1038/s41380-018-0028-2 29487402 PMC6111001

[49] Payne JL, Maguire J. Pathophysiological mechanisms implicated in postpartum depression. Front Neuroendocrinol. 2019;52:165-180. doi:10.1016/j.yfrne.2018.12.001 30552910 PMC6370514

[50] Swenson CW, DePorre JA, Haefner JK, Berger MB, Fenner DE. Postpartum depression screening and pelvic floor symptoms among women referred to a specialty postpartum perineal clinic. Am J Obstet Gynecol. 2018;218(3):335.e1-335.e6. doi:10.1016/j.ajog.2017.11.604 29229409 PMC5834372

[51] Lim G, Farrell LM, Facco FL, Gold MS, Wasan AD. Labor analgesia as a predictor for reduced postpartum depression scores: a retrospective observational study. Anesth Analg. 2018;126(5):1598-1605. doi:10.1213/ANE.0000000000002720 29239949 PMC5908733

[52] Orbach-Zinger S, Landau R, Harousch AB, . The relationship between women’s intention to request a labor epidural analgesia, actually delivering with labor epidural analgesia, and postpartum depression at 6 weeks: a prospective observational study. Anesth Analg. 2018;126(5):1590-1597. doi:10.1213/ANE.0000000000002501 28930940

[53] Bilgen S, Köner O, Türe H, Menda F, Fiçicioğlu C, Aykaç B. Effect of three different doses of ketamine prior to general anaesthesia on postoperative pain following caesarean delivery: a prospective randomized study. Minerva Anestesiol. 2012;78(4):442-449. 22240615

[54] Patel RR, Murphy DJ, Peters TJ. Operative delivery and postnatal depression: a cohort study. BMJ. 2005;330(7496):879. doi:10.1136/bmj.38376.603426.D3 15734748 PMC556158

