# Supplementary material for: Perioperative Adjunctive Esketamine for Postpartum Depression Among Women Undergoing Elective Cesarean Delivery: A Randomized Clinical Trial
Source: JAMA Netw Open. 2024 Mar 6;7(3):e240953. doi: 10.1001/jamanetworkopen.2024.0953 (PMC10918550; doi:10.1001/jamanetworkopen.2024.0953)
Supplement: Supplement 1. — Trial Protocol and Statistical Analysis Plan [file jamanetwopen-e240953-s001.pdf]

Perioperative Esketamine for Prevention of Postpartum Depression after Cesarean Delivery: A Randomized Clinical Trial

Study Protocol

Principal investigator: Prof Xiao-Dan Wu, MD

Organizer institute: Department of Anesthesiology, South branch of Fujian Provincial Hospital

Clinical site: Fujian Provincial Hospital; South branch of Fujian Provincial Hospital.

Contact: Yu Chen, MD

Start Time: January, 2022

Stop Time: January, 2023

Version of protocol: V 2.0

Date of version: November 25th, 2021

Contents

1. Background....3
2. Study purpose....4
3. Study design....4
   3.1. Type of the study....4
   3.2. Sample size calculation....4
   3.3. Participant recruitment....5
   3.4. Randomization and blinding....5
   3.5. Intervention protocol....5
   3.6. Remedial measures....6
4. Data collection....6
   4.1. Baseline data....6
   4.2. Intraoperative data....6
   4.3. Neonatal outcomes....6
   4.4. Postoperative data....6
5. Outcomes....7
6. Adverse events....7
7. Quality control....7
8. Statistical analysis....7
9. References....8

1. Background

Postpartum depression (PPD) refers to the depression disorder that occurs during the puerperium, which is a puerperal psychiatric syndrome along with postpartum dysphoria and postpartum psychosis, and it is a relatively common postpartum psychological disorder for women, with an incidence rate as high as 15% to 30%. It mainly manifests symptoms such as depressed mood, reduced interest in activities, sadness, irritability, insomnia, tiredness, decreased concentration, and even recurring suicidal thoughts, seriously affecting postpartum women's physical and mental health[1,2]. Tragically, it can even lead to suicidal thoughts, accounting for about 20% of postnatal maternal deaths[3]. In addition, PPD also adversely affects infants' behavioral, emotional, and cognitive development, causing a significant burden on families. With the increasing incidence of PPD, there has been growing interest in understanding the pathogenesis of PPD[4,5].

N-methyl-D-aspartate (NMDA) receptor antagonists have significant analgesic and sedative effects, and its classical representative drug, ketamine, is one of the critical components of clinical anesthesia and multimodal analgesia[6-8]. As the newest NMDA receptor antagonist, Esketamine is the S-enantiomer of ketamine, which has a stronger affinity for NMDA receptors and has a more potent anesthetic and analgesic activity as well as a more precise antidepressant effect compared with ketamine[9]. Therefore, in recent years, the clinical research on esketamine in the treatment of depression has been deepening, and the study by Singh et al. found that the treatment of esketamine nasal spray is a novel and reliable option for patients with refractory depression who do not respond well to standard treatment approaches[10,11]. Recent reviews have also highlighted that the introduction of esketamine is a new hope for patients with refractory depression and holds a unique and indispensable role in combating this challenging condition[12].

However, the effect of esketamine on postpartum pain-depression co-morbidity has not been definitively established. Given its dual therapeutic potential in pain management and antidepressant effects, our research team intends to conduct a prospective RCT study, taking the mothers who have undergone cesarean section to terminate pregnancy in our hospital as the research participants, and observing the effect of intravenous administration of esketamine on the analgesic effect and the occurrence of PPD after cesarean section, by collecting the perinatal data and the data from the follow-up visit in the postnatal clinic of our hospital in the 6 weeks after the delivery. We will employ statistical methods, including univariate analysis and multifactorial logistic regression analysis, to assess the effects of intravenous esketamine on the analgesic effect after cesarean section and the occurrence of PPD. This study is expected to provides clinical evidence for the prevention and treatment of postpartum depression.

2. Study purpose

To explore the effect of intravenous administration of esketamine on both the incidence of PPD and its analgesic efficacy following cesarean section.

3. Study design

3.1. Type of the study

The research is a single-center, double-blind, placebo-controlled, randomized clinical trial. The flow chart of the study is shown in Figure 1

Figure 1

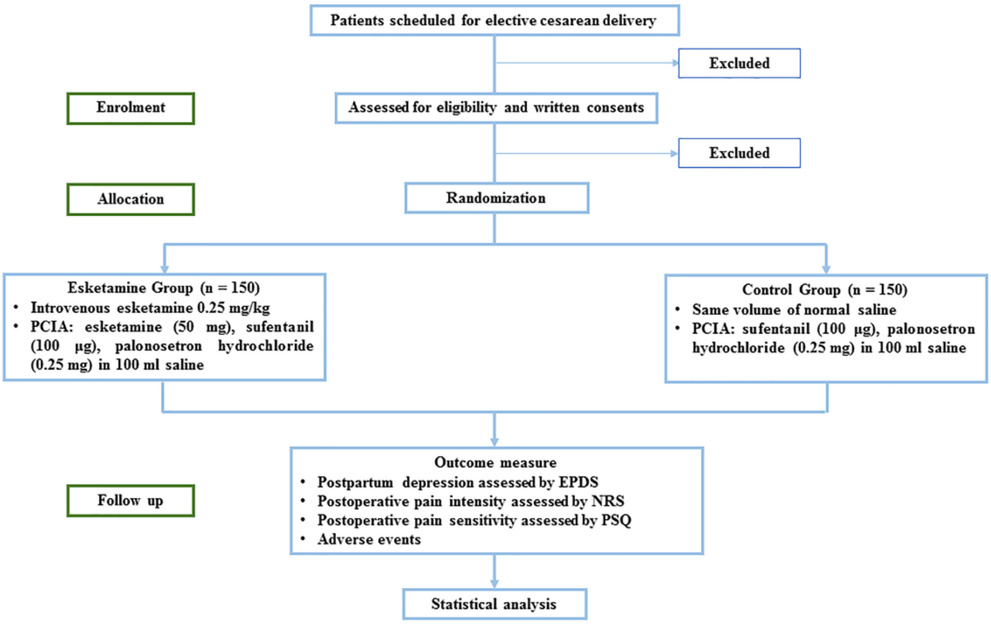

3.2. Sample size calculation

This was based on our preliminary study, in which the incidence of screened positivity for PPD on postpartum day 7, was 35% for patients who underwent cesarean deliveries. The expected effect size was subsequently calculated to detect a 50% prevalence reduction in PPD after surgery, with a 2-sided α = .05 and 90% power. The sample size required was determined to be 128 patients. Considering the possibility of loss to follow-up and consent withdrawals, 150 patients were selected for inclusion in each arm of this trial.

3.3. Participant recruitment

3.3.1. Inclusion criteria

3.3.1.1 Women aged 18 to 40 years;

3.3.1.2 American Society of Anesthesiologists (ASA) grade Ⅰ to Ⅲ classification;

3.3.1.3 Singleton pregnancy;

3.3.1.4 full-term (> 37 weeks) pregnancy;

3.3.1.5 Scheduled for elective cesarean delivery.

3.3.2. Exclusion criteria

3.3.2.1 Patients with prenatal mental disorder;

3.3.2.2 Patients with hypertension or risk of elevated intracranial pressure;

3.3.2.3 Patients with pre-eclampsia and eclampsia;

3.3.2.4 Patients with hyperthyroidism;

3.3.2.5 Patients with placenta previa, placental abruption, and placenta accreta;

3.3.2.6 Allergy to NMDA receptor antagonist;

3.3.2.7 Unable or unwilling to cooperate with the questionnaires and clinical examinations.

3.4. Randomization and blinding

In the study, an independent researcher who otherwise were not involved in the trial's execution or statistical analysis, generated a numerical random sequence using a computer. This sequence was distributed discreetly through sealed envelopes. When eligible patients were enrolled in the study, the envelopes were handed over to a nurse anesthetist, who opened them and prepared medication based on the information contained within.

3.5. Intervention protocol

3.5.1 Esketamine group: Following the delivery of the fetus, 0.25 mg/kg of esketamine diluted to 10 ml with 0.9% saline was administered intravenously. Subsequently, sufentanil (100 µg), esketamine (50 mg), and palonosetron hydrochloride (0.25 mg) dissolved in 100 ml saline were given using a patient-controlled intravenous analgesia (PCIA) device.

3.5.2 Control group: The equivalent volume of normal saline was administered

intravenously after fetus delivery. Moreover, sufentanil (100 μg) and palonosetron hydrochloride (0.25 mg) in 100 ml saline were also given using a PCIA pump.

3.6. Remedial measures

3.6.1 When the subarachnoid block fails, local anesthetic solution is incrementally added through the epidural catheter in the extradural space until the block level reaches T6.
3.6.2 Postoperatively, if the NRS pain score exceeds 5 points, patients may self-administer a bolus dose. If the NRS score remains above 4 points after 5 minutes, 50 mg of flurbiprofen axetil is administered intravenously until the NRS score is $\leqslant 3$ points.
3.6.3 The above measures will be recorded.

4. Data collection

4.1. Baseline data

Age, height, weight, pregestational comorbidity, number of gravidity and parturitions, duration of gestation, ASA classification, and Baseline EPDS score.

4.2. Intraoperative data

Duration of surgery, estimated blood loss, dosage of oxytocin, and use of vasopressors

4.3. Neonatal outcomes

Birth weight, APGAR scores at 1 min, 5 min, 10 min, Umbilical artery blood gas.

4.4. Postoperative data

4.4.1 Assessment of postpartum depressive symptoms using the Edinburgh Postnatal Depression Scale (EPDS) at the following time points: day 7, 14, 28, and 42 following delivery.
4.4.2 Evaluation of postoperative pain intensity at rest and during movement assessed with NRS pain score at designated time points: 12, 24, 48, and 72 h after surgery.
4.4.3 Analysis of postoperative pain sensitivity assessed with pain sensitivity questionnaire scores (PSQ-C) 7 days after surgery.

5. Outcomes

5.1. Primary outcomes：postpartum depressive symptoms

5.2. Secondary outcomes: postoperative pain intensity, pain sensitivity, and the recovery of postoperative gastrointestinal function

6. Adverse events

Including postoperative nausea and vomiting, as well as neuropsychiatric symptoms such as nystagmus, dizziness, headache, nightmares, and hallucinations.

7. Quality control

All research data, both in paper and electronic formats, will be kept for at least 5 years. If readers have any questions after the article's publication, they may contact the corresponding author for access to the original data. Patient information will remain anonymous, including name, ID number and telephone number. All data related to enrolled patients collected during the study period of this project will be archived and safeguarded, and all study members and study sponsors are asked to keep subject information confidential. The study protocol will be reviewed and revised by statistical experts. Investigators will adhere to pre-defined standard operating procedures, encompassing patient screening, follow-up standardization, result assessment, and data management. The Ethics Committee of Fujian Provincial Hospital will review the trial every six months.

8. Statistical analysis

The normality of variables was examined using the Kolmogorov-Smirnov test. Continuous data were presented as mean (SD) and intergroup compared using the unpaired, 2-tailed t-test or One-Way ANOVA test if distributed normally. Variables not normally distributed were reported as median (IQR) and intergroup compared using the Mann-Whitney U test. Categorical variables were presented as numbers (%) and were subjected to comparison using either the $\chi 2$ or Fisher exact test, as appropriate. Change in EPDS scores at different points of time between groups was analyzed using a mixed-effects model using repeated measures (MMRM). The model included baseline EPDS

score as a covariate, and treatment, day, and day-by-treatment interaction as fixed effects. Random intercepts and unstructured covariance structures were used to model the within-patient errors. An exploratory analysis was conducted to evaluate disparities in our primary outcome. The stratification confounders were corrected by the Cochran-Mantel-Haenszel test.

The level of significance will be established at $\alpha < 0.05$ with a two-sided test.

9. References

1. Liu Y, Zhang L, Guo N, Jiang H. Postpartum depression and postpartum post-traumatic stress disorder: prevalence and associated factors. *BMC psychiatry.* 2021;21(1):487.
2. Deligiannidis KM, Meltzer-Brody S, Gunduz-Bruce H, et al. Effect of Zuranolone vs Placebo in Postpartum Depression: A Randomized Clinical Trial. *JAMA psychiatry.* 2021;78(9):951-959.
3. Surkan PJ, Patel SA, Rahman A. Preventing infant and child morbidity and mortality due to maternal depression. *Best practice & research Clinical obstetrics & gynaecology.* 2016;36:156-168.
4. Feldman R, Granat A, Pariente C, Kanety H, Kuint J, Gilboa-Schechtman E. Maternal depression and anxiety across the postpartum year and infant social engagement, fear regulation, and stress reactivity. *Journal of the American Academy of Child and Adolescent Psychiatry.* 2009;48(9):919-927.
5. Becker M, Weinberger T, Chandy A, Schmukler S. Depression During Pregnancy and Postpartum. *Current psychiatry reports.* 2016;18(3):32.
6. Uno Y, Coyle JT. Glutamate hypothesis in schizophrenia. *Psychiatry and clinical neurosciences.* 2019;73(5):204-215.
7. Jorratt P, Hoschl C, Ovsepian SV. Endogenous antagonists of N-methyl-d-aspartate receptor in schizophrenia. *Alzheimer's & dementia : the journal of the Alzheimer's Association.* 2021;17(5):888-905.
8. Opler LA, Opler MG, Arnsten AF. Ameliorating treatment-refractory depression with intranasal ketamine: potential NMDA receptor actions in the pain circuitry representing mental anguish. *CNS spectrums.* 2016;21(1):12-22.
9. Bozymski KM, Crouse EL, Titus-Lay EN, Ott CA, Nofziger JL, Kirkwood CK. Esketamine: A Novel Option for Treatment-Resistant Depression. *The Annals of pharmacotherapy.* 2020;54(6):567-576.
10. Singh JB, Fedgchin M, Daly E, et al. Intravenous Esketamine in Adult Treatment-Resistant Depression: A Double-Blind, Double-Randomization, Placebo-Controlled Study. *Biological psychiatry.* 2016;80(6):424-431.
11. Daly EJ, Singh JB, Fedgchin M, et al. Efficacy and Safety of Intranasal Esketamine Adjunctive to Oral Antidepressant Therapy in Treatment-Resistant Depression: A Randomized Clinical Trial. *JAMA psychiatry.* 2018;75(2):139-148.
12. Molero P, Ramos-Quiroga JA, Martin-Santos R, Calvo-Sánchez E, Gutiérrez-Rojas L, Meana JJ. Antidepressant Efficacy and Tolerability of Ketamine and Esketamine: A Critical Review.

CNS drugs. 2018;32(5):411-420.
