# Supplementary material for: Perioperative Adjunctive Esketamine for Postpartum Depression Among Women Undergoing Elective Cesarean Delivery: A Randomized Clinical Trial
Source: JAMA Netw Open. 2024 Mar 6;7(3):e240953. doi: 10.1001/jamanetworkopen.2024.0953 (PMC10918550; doi:10.1001/jamanetworkopen.2024.0953)
Supplement: Supplement 2. — eTable 1. The Prevalence of Women With a Positive PPD Screening (EPDS Score ≥10) Between the Two Groups eTable 2. The Changes in EPDS Scores From Baseline Values to the End-Points of the Trial Period Between the Two Groups eTable 3. The Numeric Rating Scale Pain Scores Between the Two Groups at Different Time Points eTable 4. Postoperative Recovery Outcomes Between the Two Groups eTable 5. Adverse Events in the Study Participants eFigure. Trial Intervention Diagram [file jamanetwopen-e240953-s002.pdf]

Supplementary Online Content

Chen Y, Guo Y, Wu H, et al. Perioperative adjunctive esketamine for postpartum depression among women undergoing elective cesarean delivery: a randomized clinical trial. *JAMA Netw Open.* 2024;7(3):e240953. doi:10.1001/jamanetworkopen.2024.0953

eTable 1. The Prevalence of Women With a Positive PPD Screening (EPDS Score ≥10) Between the Two Groups
eTable 2. The Changes in EPDS Scores From Baseline Values to the End-Points of the Trial Period Between the Two Groups
eTable 3. The Numeric Rating Scale Pain Scores Between the Two Groups at Different Time Points
eTable 4. Postoperative Recovery Outcomes Between the Two Groups
eTable 5. Adverse Events in the Study Participants
eFigure. Trial Intervention Diagram

This supplementary material has been provided by the authors to give readers additional information about their work.

eTable 1. The Prevalence of Women With a Positive PPD Screening (EPDS Score ≥10) Between the Two Groups

**Outcome**	**Esketamine (n=148)**	**Control (n=150)**	**Treatment Effect (95% CI)**	***P* Value**
**Postpartum day 7**				
Positive PPD, NO. (%)	34 (23.0)	53 (35.3)	0.55 (0.33, 0.91)	0.02
**Postpartum day 14**				
Positive PPD, NO. (%)	46 (31.1)	57 (38.0)	0.74 (0.46, 1.19)	0.23
**Postpartum day 28**				
Positive PPD, NO. (%)	53 (35.8)	55 (36.7)	0.96 (0.60, 1.55)	0.90
**Postpartum day 42**				
Positive PPD, NO. (%)	50 (33.8)	56 (37.3)	0.85 (0.53, 1.36)	0.55

Abbreviations: PPD, postpartum depression; EPDS, Edinburgh Postnatal Depression Scale.

Data reported as the number of patients (%) were compared using the either the χ^2 or Fisher exact tests.

© 2024 Chen Y et al. *JAMA Network Open.*

eTable 2. The Changes in EPDS Scores From Baseline Values to the End-Points of the Trial Period Between the Two Groups

	Change from baseline, mean (SD)		Difference of LS, means (SE)	95% CI	*P* Value
	Esketamine (n=148)	Control (n=150)			
**Primary efficacy endpoint**					
postpartum day 7	3.4 (4.1)	4.6 (3.4)	-1.17 (0.44)	-0.31 to -2.04	0.008
**Secondary efficacy endpoint**					
postpartum day 14	3.6 (4.5)	3.2 (3.8)	0.32 (0.49)	-0.63 to 1.28	0.51
postpartum day 28	3.5 (4.8)	3.3 (4.1)	0.24 (0.52)	-0.78 to 1.26	0.64
postpartum day 42	3.0 (4.4)	3.1 (4.2)	-0.06 (0.49)	-1.03 to 0.91	0.90

Abbreviations: EPDS, Edinburgh Postnatal Depression Scale (ranges from 0 to 30, a higher score indicates a more severe condition); LS, least-squares.

Mixed model for repeated measures (MMRM) analysis with changes from the baseline values as the response variable and the fixed-effect model terms for treatment (esketamine and control), day and day-by-treatment interaction, and the baseline EPDS scores used as the covariate values.

© 2024 Chen Y et al. *JAMA Network Open.*

eTable 3. The Numeric Rating Scale Pain Scores Between the Two Groups at Different Time Points

**Time-points**	**Esketamine (n=148)**	**Control (n=150)**	**Treatment Effect (95% CI)**	***P* Value**
**NRS score at rest**				
Postoperative 12 h	2.0 [1.0, 2.0]	2.0 [1.0, 2.0]	Median difference, 0 [0, 0]	0.42
Postoperative 24 h	1.0 [0.0, 2.0]	1.0 [0.0, 2.0]	Median difference, 0 [0, 0]	0.47
Postoperative 48 h	0.0 [0.0, 0.0]	0.0 [0.0, 0.0]	Median difference, 0 [0, 0]	0.06
Postoperative 72 h	0.0 [0.0, 0.0]	0.0 [0.0, 0.0]	Median difference, 0 [0, 0]	0.16
**NRS score on movement**				
Postoperative 12 h	3.0 [2.0, 3.0]	2.0 [2.0, 3.0]	Median difference, 0 [0, 0]	0.31
Postoperative 24 h	4.0 [3.0, 4.0]	4.0 [3.0, 4.0]	Median difference, 0 [0, 0]	0.86
Postoperative 48 h	4.0 [3.0, 4.0]	4.0 [3.0, 4.0]	Median difference, 0 [0, 0]	0.35
Postoperative 72 h	3.0 [2.0, 3.0]	3.0 [3.0, 3.5]	Median difference, 0 [0, 0]	0.03

Abbreviations: NRS, numeric rating scale (ranges from 0 to 10, with 0 indicating no pain and 10 indicating the worst pain).

Data presented as medians [IQRs] were compared using the Mann-Whitney test.

© 2024 Chen Y et al. *JAMA Network Open.*

eTable 4. Postoperative Recovery Outcomes Between the Two Groups

**Outcome**	**Esketamine (n=148)**	**Control (n=150)**	**Treatment Effect (95% CI)**	***P* Value**
Time to first flatus	1.0 [0.5, 2.0]	1.0 [0.5, 2.0]	Median difference, 0 [0, 0]	0.53
Time to first defecation	2.0 [2.0, 3.0]	2.0 [2.0, 3.0]	Median difference, 0 [0, 0]	0.49
Length of hospital stay	4.0 [3.0, 4.0]	4.0 [3.0, 4.0]	Median difference, 0 [0, 0]	0.14

Data presented as medians [IQRs] were compared using the Mann-Whitney test.

© 2024 Chen Y et al. *JAMA Network Open.*

eTable 5. Adverse Events in the Study Participants

Adverse events	Esketamine (n=148)	Control (n=150)	*P* value
Nausea or vomiting, NO. (%)	12 (8.1)	16 (10.7)	0.55
Dizziness, NO. (%)	1 (0.7)	4 (2.7)	0.37
Headache, NO. (%)	1 (0.7)	2 (1.3)	> 0.99
Somnolence, NO. (%)	5 (3.4)	3 (2.0)	0.50
Hallucination, NO. (%)	0 (0.0)	0 (0.0)	NA
Nightmare, NO. (%)	0 (0.0)	0 (0.0)	NA
Nystagmus, NO. (%)	0 (0.0)	0 (0.0)	NA

Abbreviations: NA, not applicable.

Data reported as the number of patients (%) were compared using either the χ^2 or Fisher exact tests.

© 2024 Chen Y et al. *JAMA Network Open.*

eFigure. Trial Intervention Diagram

A. The intervention of the control group. B. The intervention of the esketamine group.

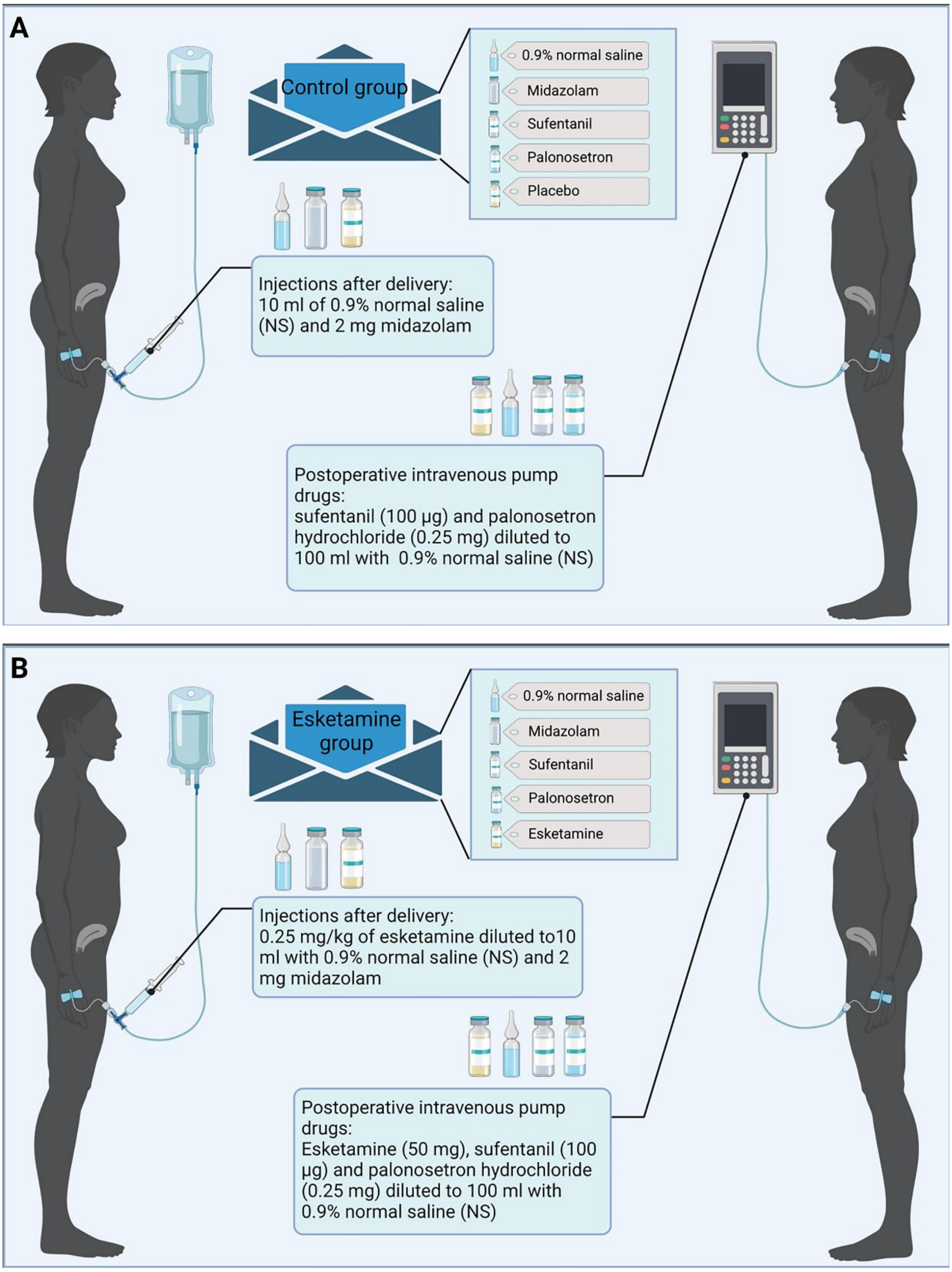

© 2024 Chen Y et al. *JAMA Network Open.*
