# Supplementary material for: Perioperative Adjunctive Esketamine for Postpartum Depression Among Women Undergoing Elective Cesarean Delivery: A Randomized Clinical Trial
Source: JAMA Netw Open. 2024 Mar 6;7(3):e240953. doi: 10.1001/jamanetworkopen.2024.0953 (PMC10918550; doi:10.1001/jamanetworkopen.2024.0953)
Supplement: Supplement 3. — Data Sharing Statement [file jamanetwopen-e240953-s003.pdf]

Data Sharing Statement

Chen. Perioperative Adjunctive Esketamine for Postpartum Depression Among Women Undergoing Elective Cesarean Delivery. *JAMA Netw Open*. Published March 06, 2024. doi:10.1001/jamanetworkopen.2024.0953

Data
Data available: No
